# Endophyte species influence the biomass production of the native grass *Achnatherum sibiricum* (L.) Keng under high nitrogen availability

**DOI:** 10.1002/ece3.2566

**Published:** 2016-11-10

**Authors:** Xia Li, Yong Zhou, Wade Mace, Junhua Qin, Hui Liu, Wei Chen, Anzhi Ren, Yubao Gao

**Affiliations:** ^1^ Department of Plant Biology and EcologyCollege of Life SciencesNankai UniversityTianjinChina; ^2^ AgResearch LtdGrasslands Research CentrePalmerston NorthNew Zealand; ^3^ Present address: College of Life SciencesHebei UniversityBaodingChina

**Keywords:** *Achnatherum sibiricum*, endophyte species, native grass, nitrogen, plant maternal genotype

## Abstract

Research on the interaction of endophytes and native grasses normally takes infection status into account, but less often considers the species of endophyte involved in the interaction. Here, we examined the effect of endophyte infection, endophyte species, nitrogen availability, and plant maternal genotype on the performance of a wild grass, *Achnatherum sibiricum*. Six different *Epichloë*‐infected maternal lines of *A*.* sibiricum* were used in the study; three lines harbored *Epichloë gansuensis* (*Eg*), while three lines harbored *Epichloë sibirica* (*Es*). These endophytes are vertically transmitted, while *Eg* also occasionally produces stromata on host tillers. We experimentally removed the endophyte from some ramets of the six lines, with the infected (E+) and uninfected (E−) plants grown under varying levels of nitrogen availability. *Eg* hosts produced more aboveground biomass than *Es* hosts only under high nitrogen supply. Endophyte species did not show any influence on the maximum net photosynthetic rate (*P*_max_), photosynthetic nitrogen use efficiency, or total phenolics of *A. sibiricum* under all nitrogen conditions. However, the plant maternal genotype did influence the *P*_max_ and shoot biomass of *A*.* sibiricum*. Our results show that endophyte species influenced the shoot biomass of *A. sibiricum*, and this effect was dependent on nitrogen supply. As with most coevolutionary interactions, *A. sibiricum* that harbored *Eg* and *Es* may show pronounced geographic variation in natural habitats with increased nitrogen deposition. In addition, stroma‐bearing endophyte (*Eg*) provides positive effects (e.g., higher biomass production) to *A. sibiricum* plants during the vegetative growth stage.

## 1 Introduction

Plants form and maintain numerous symbioses with a variety of microbes in natural communities (Dighton, White, & Oudemans, 2005; Franche, Lindström, & Elmerich, 2009; Hardoim, van Overbeek, & van Elsas, 2008). To date, approximately 270,000 plant species on earth are estimated to harbor approximately 13.6 million unique fungal species (Dreyfuss & Chapela, 1994). For example, arbuscular mycorrhizal fungi (AMF) and dark septate endophytes (DSEs) colonize plant roots. Increased water and nutrient uptake as well as stress tolerance in exchange for host photosynthates are among the many benefits obtained by infected hosts (Augé, Kubikova, & Moore, 2001; Smith & Read, 2008). In addition, fungal endophytes are hyperdiverse community that make up a major component of plant microbiota.

Over the past few decades, much has been learned about the unique symbiotic interaction between *Epichloë* endophytes (both teleomorph‐type and anamorph‐type endophytes) and cool‐season grasses (Clay, 1990; Glenn, Bacon, Price, & Hanlin, 1996; Rostás, Cripps, & Silcock, 2015; Schardl, Leuchtmann, & Spiering, 2004; Shukla, Hager, Yurkonis, & Newman, 2015). *Epichloë* endophytes grow asymptomatically in the intercellular spaces of the aboveground plant parts for at least part of their life cycle (Rodriguez, White, Arnold, & Redman, 2009). The symbiotic interaction of the host grasses with endophytes has been shown to be strongly mutualistic in studies using agronomic grasses, such as tall fescue and perennial ryegrass (Assuero et al., 2000; Clay, 1990; Clay & Schardl, 2002; Hesse et al., 2003; Rudgers, Koslow, & Clay, 2004).

However, accumulating evidence suggests that the degree of the mutual benefit is conditional on nutrient factors, in particular nitrogen (N) availability in soils (Saikkonen, Lehtonen, Helander, Koricheva, & Faeth, 2006). As one of the most important limiting resources for plant growth, nitrogen affects all levels of plant function, from metabolism to biomass production and growth (Crawford, 1995; Marschner, 1995; Stitt & Krapp, 1999). Nitrogen is also a constituent of alkaloids in infected plants. For example, *Epichloë* endophytes may produce N‐rich secondary metabolites, such as loline alkaloids that can constitute up to 2% of the dry weight of the plant (Blankenship et al., 2001; De‐wen, Wang, Patchett, & Gooneratne, 2006). Elevated nitrogen supply can alter plant nutrient allocation and may affect nutrient exchange in ways that impact the production of N‐based mycotoxins and carbon energy sources for endophytic growth. Höft, Verpoorte, and Beck (1996) also found a variable relationship between nitrogen and alkaloid production/accumulation. However, published reports of the effects of N availability on grass–endophyte associations are inconsistent. Arachevaleta, Bacon, Hoveland, and Radcliffe (1989) reported that the beneficial growth responses of tall fescue from one clone to endophyte depend on a high nitrogen supply in the environment. In another study using *Lolium perenne* seeds from a single plant (Ren, Gao, Wang, Wang, & Zhao, 2009), endophyte‐infected (E+) plants maintained significantly higher above‐ and belowground biomass than endophyte‐free (E−) hosts under high‐nitrogen conditions, while the positive effect of endophyte infection disappeared under low‐nitrogen conditions. These studies suggest that with nitrogen limitation, endophytes may have neutral or negative effects on the performance of host plants. However, several works have shown the opposite results. For example, in a low‐nitrogen environment, the presence of an endophyte has been found to improve plant performance (such as vegetative growth and biomass accumulation) in three genotypes of *L. perenne* (Lewis, 2004; Ravel, Courty, Coudret, & Charmet, 1997).

In addition to environmental factors, Cheplick (1997) proposed that the benefits of infection may also depend on the genotype of the host plants. In the case of tall fescue and *L. perenne*, the effects of the plant genotype and interactions with endophyte infection were reported to have significant effects on the performance (such as tiller numbers, specific leaf area, nutrient content and biomass) of host plants (Belesky & Fedders, 1996; Cheplick, 1997, 1998, 2004, 2007; Cheplick & Cho, 2003; Hesse et al., 2004; Rahman & Saiga, 2005). In a 3‐year common garden study of nine genotypes of *L. perenne*, Cheplick (2008) even found that the effects of the host genotype may outweigh the effects of fungal endophyte infection on the number of tillers and biomass of *L. perenne*. Nonetheless, many studies of host genotype interaction with endophyte infection only compared the performance of E+ and E− hosts, without considering endophyte species or genotypes (West, Popay, & Thom, 2007). When studying endophyte species of agronomic importance, researchers often use host plants inoculated with selected specific endophyte genotypes. Tall fescue infected by one of two distinct endophyte strains designated CTE and AR‐584 were employed in a glasshouse experiment as reported in Rúa, McCulley, and Mitchell (2013). The total biomass of the host plants when infected with CTE was significantly greater than that of E− hosts, while the presence of AR‐584 appeared to not be significant. Another inoculation experiment employing two cultivars of tall fescue was performed using two endophyte genotypes (wild type, KY31, designated type, AR501) of *Epichloë coenophiala*. The results showed that there was a significant cultivar × endophyte strain interaction for most measured variables (such as the tiller number, growth rate, and photosynthesis; Assuero et al., 2000).

In comparison with agricultural grasses, there are more endophyte species in naturally occurring native grass populations. So far, multiple endophyte species have been reported from *Festuca arizonica* (Sullivan & Faeth, 2004), *Achnatherum inebrians* (Li, Nan, Paul, Dapprich, & Liu, 2004; Moon et al., 2007), *Achnatherum sibiricum* (Zhang et al., 2009), *Bromus auleticus* (Iannone, Pinget, Nagabhyru, Schardl, & De Battista, 2012), *Hordelymus europaeus* (Leuchtmann & Oberhofer, 2013), and *Achnatherum robustum* (Shymanovich et al., 2015). For example, Leuchtmann and Oberhofer (2013) found that *H. europaeus* was infected by one of six endophyte species in the grass population. Recent studies about the influence of endophyte infections on native grasses, however, have mostly compared E+ and E− hosts (Faeth, Bush, & Sullivan, 2002; Faeth, Helander, & Saikkonen, 2004; Gibert & Hazard, 2013; Gundel et al., 2013; Morse, Day, & Faeth, 2002; Ren et al., 2011; Saari, Helander, Faeth, & Saikkonen, 2010; Zhang, Fan, Li, & Nan, 2010). Far less is known about the influence of endophyte species on wild host grasses, and the results seem to be variable and inconsistent. For example, Hamilton, Dowling, and Faeth (2010) found that *F. arizonica*, infected with one of two endophyte species (*E. tembladerae* and *E. huerfana*), show different performances in a controlled experiment. Infection by *E. tembladerae* resulted in significantly higher total biomass and host survival compared to hosts infected with *E. huerfana*. Iannone et al. (2012) also found that *B. auleticus* infected with *Epichloë pampeana* produced more biomass than plants infected with *E. huerfana*. However, in other cases, the endophyte species appeared to have neutral effects on the vegetative growth of *H. europaeus* (Oberhofer & Leuchtmann, 2014).

The endophyte utilizes photosynthetic carbon and additional elements, particularly nitrogen, from its host to carry out the metabolic processes necessary for its survival and growth (Christensen & Bennett, 2002). According to previous reports, endophyte infection can have significant effects on host photosynthetic parameters, and these effects are contradictory (Belesky Devine, Pallas, & Stringer 1987; Morse et al., 2002). Belesky et al. (1987) reported that E+ plants showed decreased rate of photosynthesis. On the other hand, Amalric, Sallanon, Monnet, Hitmi, Coudret, & Amalric (1999) demonstrated higher photosynthetic rates under drought stress, which is similar to the study that Marks and Clay (1996) conducted at high temperatures. In addition to these results, the endophyte haplotype appears to override the infection status in some cases, at least in several physiological measures (e.g., Pn; Morse, Faeth, & Day, 2007). Thus, we expected that *A. sibiricum* infected with different endophyte species may have varied photosynthesis‐related mechanisms.

*Achnatherum sibiricum* (L.) Keng, infected with two different endophytes, *E. gansuensis* and *E*.* sibirica* in its native populations, is a caespitose perennial grass distributed in northern China (Wei, Gao, Li, Xu, & Ren, 2006; Zhang et al., 2009). In previous studies by our laboratory, five *A. sibiricum* populations were sampled and the distribution and abundance of endophytes were documented. In total, 438 fungal isolates were obtained, but the phenomenon of double *Epichloë* infections in *A. sibiricum* has never been observed. The overall abundance of the two dominant endophyte species remained constant in all investigated populations (92%–95%; Zhang et al., 2009). Both *E*.* gansuensis* and *E*.* sibirica* can be vertically transmitted from the maternal host to the offspring by seed. However, conidia stromata have occasionally been observed on culms of *A*.* sibiricum* plants harboring *E*.* gansuensis*. The conidia derived from the stromata can give rise to mycelia that in turn infect germinating endophyte‐free seeds, which indicated its ability for horizontal transmission (Li et al., 2015). Furthermore, it has been reported that *E*.* gansuensis* naturally infects both *A*.* sibiricum* and *A*.* inebrians* (Li et al., 2004; Zhang et al., 2009), while *E*.* sibirica* naturally infects only *A*.* sibiricum* (Zhang et al., 2009). In contrast to *A*.* inebrians*,* A*.* sibiricum* plants have no obvious herbivore deterrence according to previous observations (Zhang et al., 2009). Given the above, *A*.* sibiricum* is considered to be an excellent research material to study the effect of endophyte species on host plants. In a previous study, we have compared the performance of E+ and E− *A. sibiricum* under different nitrogen availability levels. The results showed that *A. sibiricum*–*Epichloë* associations were conditional on both N and P availability, but more conditional on N than on P. The aim of this work was to characterize the effect of the endophyte species on the performance of *A. sibiricum*, and also to consider the effects of the maternal genotype of the host as well as the nitrogen availability in the soil. We addressed the following questions: (1) is the mutual effect between the endophyte and native grass related to the species of endophyte? If so, (2) does the influence of the endophyte species depend on the nitrogen availability? (3) Does the host maternal genotype affect the symbiosis of the endophyte and native grass?

## 2 Materials and Methods

### 2.1 Study system

The frequency of endophyte infections in wild populations of *Achnatherum sibiricum* is usually high (86%–100%; Wei et al., 2006). Seeds of *A. sibiricum* in the present study were collected from natural populations in National Hulunber Grassland Ecosystem Observation and Research Station (119.67°E, 49.10°N) in northeast China. As *A. sibiricum* is a cross‐pollinated grass, we hereafter refer to the selected maternal seed sources as maternal plant genotypes. Within this population, 200 maternal plants were randomly selected in 2012, and a distance of at least 5 m was maintained between the sampled plants. Seeds were collected from the sampled plants and stored at 4°C. The infection rate was determined using the aniline blue staining method (Latch, Christensen, & Samuels, 1984). First, 100 seeds were randomly selected for the detection of the infection rate. Second, the other 100 randomly selected seeds were planted in 10 pots (1 m × 1 m) in the field. After 3 months, the infection rate was also determined. The results all showed that the endophyte infection frequency was 100%.

Five seeds from the same maternal plant were randomly used for the isolation and observation of endophytes. Seeds from each sample were surface‐sterilized by placing them into a 50% sulfuric acid solution for 20 min, rinsing in sterile water, soaking for 20 min in a 3% sodium hypochlorite solution, and finally rinsing three times in sterile water. Then, the surface‐sterilized seeds were placed on Petri dishes containing a potato dextrose agar (PDA) medium. The Petri dishes were incubated at 25°C for 3 weeks. After purification, two types of endophyte were observed: *E*.* gansuensis* and *E*.* sibirica* based on their colony morphology which had been reported by Zhang et al. (2009). We used seeds from six naturally infected maternal plants (G1–G6) from the same population. Among them, three of the six naturally infected plants used in our experiments (G1–G3) harbored *E*.* gansuensis* (termed *Eg*) and the other three (G4–G6) harbored *E*.* sibirica* (termed *Es*). The seeds from G1 to G6 were all divided into two parts and stored at 4°C (endophyte‐infected, E+) or underwent a 30‐day 60°C heat treatment (endophyte‐removed, E−). In our previous study, we found that this high temperature treatment had no significant effect on the germination rate, germination potential, or germination index of the seeds of *A*.* sibiricum* (Li, Han, Ren, & Gao, 2010).

### 2.2 Experimental design

E+ and E− seeds from the six maternal plants were planted in individual pots under contrasting nitrogen (N) availability regimes to test the effect of the endophyte infection, endophyte species, nitrogen availability, and maternal genotype of the host plant on the relative performance in terms of growth, photosynthetic physiology, and biomass production of *A*.* sibiricum*. Nine E+ and E− seeds of six genotypes were sown in plastic pots (28 cm × 22 cm) filled with 4 kg of sterile sand on 6th May 2013. After 40 days, six well‐grown seedlings were kept for the nitrogen treatment. The seedlings were randomly placed into each of the two nitrogen treatments: high nitrogen availability (HN) and low nitrogen availability (LN). There were three replicates per treatment group. The experiment lasted for 85 days and was carried out in the campus experimental field at Nankai University, Tianjin.

The nutrients were supplied by the addition of a modified Hoagland nutrient solution. The composition of the nutrient solution was 5.0 mmol/L CaCl_2_, 5.0 mmol/L KCl, 2.5 mmol/L MgSO_4_·7H_2_O, 2.0 mmol/L KH_2_PO_4_, 29 μmol/L Na_2_‐EDTA, 20 mmol/L FeSO_4_·7H_2_O, 45 mmol/L H_3_BO_3_, 6.6 mmol/L MnSO_4_, 0.8 mmol/L ZnSO_4_·7H_2_O, 0.6 mmol/L H_2_MoO_4_, 0.4 mmol/L CuSO_4_·5H_2_O, and pH 6.0 ± 0.1. Nitrogen was added in the form of NH_4_NO_3_, which was delivered as 0.1 m mol N (LN) or 1 m mol N (HN), respectively. The total set concentrations of the N treatments were applied according to the lower and upper limits in the natural habitat of *A*.* sibiricum* (Liu, 2004). During the experiment, 0.8 L of nutrient solution was added once a week to each pot, a total of 15 times. These pots were all located in the experimental field at Nankai University, with transparent flashing above them. Plants were subjected to ambient light and temperature regimes. The positions of the pots were randomly rotated each week to minimize location effects.

### 2.3 Photosynthesis and gas exchange parameters

At the end of the treatments, gas exchange measurements were conducted on sunny and windless days. The second youngest fully expanded leaf in a pot was measured with a LI‐COR 6400 infrared gas analyzer (LI‐Cor, Lincoln, NE, USA). The same leaf was also used to measure the specific leaf weight and nitrogen content (N%). We developed light response curves for three randomly selected *A*.* sibiricum* leaves and determined the light saturation point (LSP) per plot using a LI‐COR 6400. Under 400 μmol mol^−1^ CO_2_, the maximum net photosynthetic rate (*P*_max_) was then measured at a PPFD (1,000 μmol m^−2^ s^−1^) of the LSP in the plot (Chen, Yu, Chen, & Xu, 2006).

The nitrogen content in the leaves was measured using a vario MACRO CHN analyzer. The photosynthetic nitrogen use efficiency (PNUE) was calculated as the ratio of *P*_max_ to the area‐based leaf nitrogen concentration (Hikosaka, 2004).

### 2.4 Leaf mass per area (LMA) and total phenolic concentration

On 5th August, four fully expanded leaves per pot, growing on vegetative tillers, were oven‐dried at 60°C. They were weighed separately to determine the LMA. At the same time, three fully expanded leaves per pot were randomly collected and placed into an ice box. The total concentration of phenolics was immediately analyzed according to the method of Malinowski, Belesky, Hill, Baligar, and Fedders (1998).

### 2.5 Biomass production

At the end of the period, all of the tillers in each pot were cut off at ground level on 10th September. No reproductive shoots or stromata were observed on the tillers of the plant. The tillers were placed in an oven at 105°C for 30 min, oven‐dried at 80°C for 24 h, and then weighed to determine the aboveground biomass.

### 2.6 Endophyte alkaloid analysis

The endophyte alkaloid concentration was measured in leaf sheaths at the middle of the trial. Two tillers per plant in each pot were cut at ground level on 7th August 2013. The leaf sheaths collected from each pot were combined into a bulk sample and temporarily stored at −20°C. Prior to endophyte alkaloid analysis, bulk samples were ground in liquid nitrogen and then immediately freeze‐dried for 24 hr. Aliquots of 50 mg of the dried and ground material were analyzed for ergovaline and peramine using the methods described by Moore, Pratley, Mace, and Weston (2015). The limit of quantitation for ergovaline and peramine is 0.1 μg g^−1^.

### 2.7 Stromata observation

After they were cut off, the host plants in each pot were watered every 2 weeks until winner irrigation. Then, the pots were buried in the earth at ground level and covered with plastic transparent film. The next spring, 800 ml of distilled water was added into each pot on 12th March. After 2 weeks, 0.5L of LN and HN nutrient solutions were added into each pot. The formation of stromata was recorded from 1st April to 30th May.

### 2.8 Data analysis

Data analysis was conducted according to Morse et al. (2007). First, an initial multivariate analysis of variance (MANOVA) was used to examine the effect of infection (E+ or E−), maternal plant genotype, and nitrogen availability treatment effects on growth parameters and biomass production of *A. sibiricum* (SYSTAT 13.0). The plant maternal genotype was a nested factor within endophyte species because each plant half‐sib genotype was associated with only one of the two endophytes. As to whether the endophyte effect depends on nitrogen availability, we expect a significant interaction between the nitrogen treatment and endophyte presence. As to whether the host maternal genotype affects the symbiosis of the endophyte and native grass, we expect a significant infection of the host maternal genotype. Second, under question 1, to assess the effect of endophyte species on growth parameters and biomass production, we conducted ANOVA (SPSS 21.0) with only E+ plants because endophyte‐removed plants had no endophyte species associated with them.

## 3 Results

### 3.1 Photosynthesis and Photosynthetic nitrogen use efficiency (PNUE)

**Figure 1:**
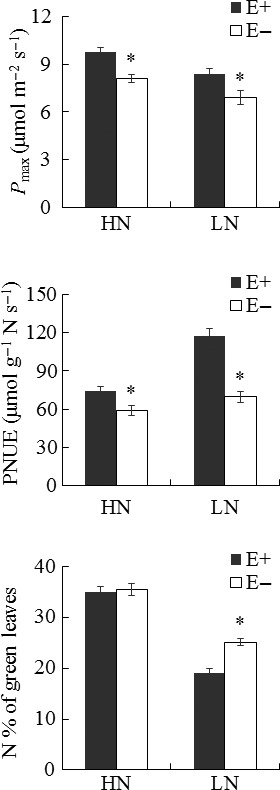
Photosynthetic parameters of endophyte‐infected (E+) and endophyte‐free (E−) *Achnatherum sibiricum* under various nitrogen availability levels. *P*_max_ is the maximum net photosynthetic rate, and PNUE is the photosynthetic nitrogen use efficiency. HN, high nitrogen availability: LN, low nitrogen availability. *Asterisks* denote significance at *p *<* *.05

**Figure 2:**
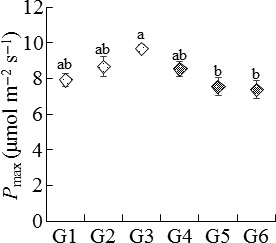
Effect of maternal plant genotype on the maximum net photosynthetic rate (*P*_max_) of *Achnatherum sibiricum*. G1–G3 are three naturally infected maternal plants that harbored *Epichloë gansuensis*, and G4–G6 are the other three naturally infected maternal plants that harbored *Epichloë sibirica*. Different letters denote significance at *p *<* *.05

There was a significant two‐way interaction between the nitrogen treatment and endophyte infection for the photosynthetic parameters of *A. sibiricum* (Table 1, Figure 1). Under both HN and LN conditions, the *P*_max_ (*p *< .001, *p *= .005) and PNUE (*p *= .006, *p *< .001) of E+ plants were significantly higher than those of E− plants. Specifically, under LN conditions, E+ plants had a significantly lower N% while they had a higher *P*_max_ than E− plants. In addition, the plant maternal genotype (nested within endophyte species) affected the *P*_max_ of host plants (*p *= .002; Table 1, Figure 2).

**Table 1:** Analysis of variance for the effects of nitrogen availability, endophyte infection, and maternal plant genotype on photosynthetic parameters, aboveground biomass, total phenolics, and specific leaf weight (LMA) of different *Achnatherum sibiricum*–endophyte symbionts

Source^a^	*df*	*P*_max_ b		PNUE^c^		Nitrogen content		Aboveground biomass		Total phenolic concentration		LMA	
		*F*	*p*	*F*	*p*	*F*	*p*	*F*	*p*	*F*	*p*	*F*	*p*
N	1	20.859	**<.001**	39.444	**<.001**	230.577	**<.001**	1234.453	**<.001**	6.089	**.017**	0.003	.958
E	1	33.465	**<.001**	54.922	**<.001**	13.865	**<.001**	19.963	**<.001**	39.826	**<.001**	4.048	**.049**
PG(EG)	4	5.038	**.002**	0.924	.457	2.397	.062	2.983	**.027**	1.688	.167	0.645	.633
E × N	1	0.145	.705	14.308	**<.001**	10.705	**.002**	4.012	.05	0.621	.434	0.124	.726
N × PG(EG)	4	1.281	.289	0.78	.543	0.639	.637	4.789	**.002**	1.574	.195	2.466	.056
E × PG(EG)	4	1.342	.267	4.197	**.005**	2.82	**.034**	0.629	.644	1.034	.399	1.93	.119
E × N × PG(EG)	4	1.599	.188	0.489	.744	2.3	.071	2.277	.073	1.088	.372	0.322	.862

a N nitrogen availability, E endophyte infection status, PG (EG) maternal plant genotype (nested within endophyte species).

b *P*_max_ maximum net photosynthetic rate.

c PNUE photosynthetic nitrogen use efficiency.

Significant *p*‐values are in bold print (*p *< .05).

The endophyte species did not affect the photosynthesis parameters of *A*.* sibiricum*. For host plants infected with different endophytes (*Es* and *Eg*), the N%, *P*_max_, and PNUE of leaves did not show any differences under all treatments (Table 2).

**Table 2:** Analysis of variance for the effects of endophyte species and nitrogen availability on photosynthetic parameters and aboveground biomass of *Achnatherum sibiricum*

Source^a^	*df*	*P*_max_ b		PNUE^c^		Nitrogen content (%)		Aboveground biomass	
		*F*	*p*	*F*	*p*	*F*	*p*	*F*	*p*
EG	1	4.153	.050	3.084	.089	0.066	.799	7.089	**.012**
N	1	12.373	**.001**	38.970	**<.001**	139.774	**<.001**	1196.366	**<.001**
EG × N	1	0.094	.761	2.180	.150	1.264	.269	2.116	.155

a EG endophyte species, N nitrogen availability.

b *P*_max_ maximum net photosynthetic rate.

c PNUE photosynthetic nitrogen use efficiency.

Significant *p*‐values are in bold print (*p *<* *.05).

### 3.2 Biomass production

**Figure 3:**
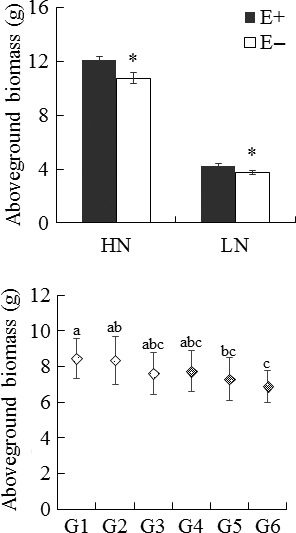
Effect of endophyte infection and maternal plant genotype on aboveground biomass of *Achnatherum sibiricum*. E+ endophyte‐infected *Achnatherum sibiricum*, E− endophyte‐free *Achnatherum sibiricum*. HN, high nitrogen availability: LN, low nitrogen availability. G1‐G3 are three naturally infected maternal plants that harbored *Epichloë gansuensis*, and G4‐G6 are the other three naturally infected maternal plants that harbored *Epichloë sibirica*. An *Asterisk* denotes significance at *p *< .05. Different letters denote significance at *p *< .05

Endophyte infection significantly improved the aboveground biomass production of *A. sibiricum*. Under HN conditions, E+ plants had a higher shoot biomass than E− plants (*p *= .005). Although the positive effect was weaker (*p *= .019), endophyte‐infected plants did show a higher biomass under LN conditions (Table 1, Figure 3). The plant maternal genotype (nested within an endophyte haplotype; *p *= .027), as well as the nitrogen treatment (*p *< .001), also affected the aboveground biomass of host plants.

**Figure 4:**
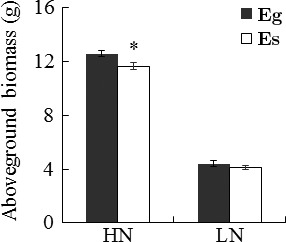
Aboveground biomass of different *Achnatherum sibiricum*–endophyte symbionts. *Eg Achnatherum sibiricum* harbored *Epichloë gansuensis*, and *Es Achnatherum sibiricum* harbored *Epichloë sibirica*. HN, high nitrogen availability: LN, low nitrogen availability. *Asterisks* denote significance at *p *< .05

At the same time, the interaction of endophyte species with the nitrogen treatment affected the biomass production of *A*.* sibiricum*. Plants infected with the *Eg* endophyte showed a higher shoot biomass than those harboring the *Es* endophyte under HN conditions (*p *= .023; Table 2, Figure 4). However, this significant effect of the endophyte species did not appear under LN conditions (*p *= .309; Table 2, Figure 4).

### 3.3 Total phenolic concentration and leaf mass per area (LMA)

**Figure 5:**
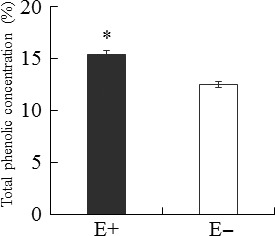
Total phenolic concentration of endophyte‐infected (E+) and endophyte‐free (E−) *Achnatherum sibiricum*. *Asterisks* denote significance at *p *< .05

**Figure 6:**
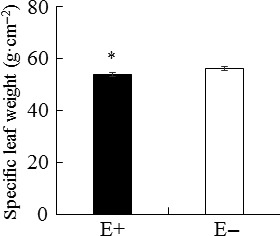
Specific leaf weight of endophyte‐infected (E+) and endophyte‐free (E−) *Achnatherum sibiricum*. *Asterisks* denote significance at *p *< .05

The total phenolic concentration and LMA of host leaves were significantly affected by endophyte infection, but not by endophyte species (Tables 1, 2). Under both HN and LN treatments, the total phenolic content (Figure 5) and LMA (Figure 6) of E+ plants were significantly higher (*p *< .001, *p *< .001) than those of E− plants. Meanwhile, these two parameters did not vary among the six maternal plant genotypes (Table 1).

### 3.4 Endophyte alkaloid production

No ergovaline was detected in any of the sampled plants. Some samples (3 of 27) showed low (<0.6 mg g^−1^) peramine production with no peramine detected in any other samples.

### 3.5 Stromata observation

On April 15th, white conidial stromata developed occasionally on tillers of *A. sibiricum* that harbored *E*.* gansuensis*. Three stromata were found on three *A. sibiricum* plants under high nitrogen treatment. The stromata persisted for 35 days on stems, but did not turn yellow.

## 4 Discussion

### 4.1 Does the mutual effect between endophyte and native grass relate to the species of endophyte?

Previous studies of the effects of endophytes on native grasses mostly considered the infection status—whether host grasses are infected or not. The existing results of the relationship are highly variable, ranging from mutualism (Brem & Leuchtmann, 2001; Crawford, Land, & Rudgers, 2010; Iannone et al., 2012; Nan, 1996; Ren et al., 2011; Rudgers & Swafford, 2009) to parasitism or commensalism (Faeth & Sullivan, 2003; Faeth et al., 2004; Hamilton & Faeth, 2005; Jani, Faeth, & Gardner, 2010; Morse et al., 2002; Saikkonen, Helander, Faeth, Schulthess, & Wilson, 1999). Generally, native grasses harbor a wider range of endophyte species compared with agronomic grasses (Iannone et al., 2012; Leuchtmann & Oberhofer, 2013; Shymanovich et al., 2015; Sullivan & Faeth, 2004; Zhang et al., 2009). For example, Leuchtmann and Oberhofer (2013) found that *H*.* europaeus* can harbor one of six endophyte species. Sullivan and Faeth (2004) also indicated that *Festuca arizonica* can naturally host one of at least two types of endophyte. It has been reported that the genetic variation in *Epichloë* endophytes can potentially influence the outcomes of interaction. Different *Epichloë* strains that have been intentionally inoculated into agronomic grasses may alter the growth and physiological properties of the host (Assuero et al., 2000). In that case, we may wonder whether the endophyte species will influence the outcome of the interactions of the endophytes with the host grasses. A previous study was conducted by Iannone et al. (2012) using *B*.* auleticus* plants infected with two types of endophyte (*E*.* pampeana* and *E*.* tembladerae*). When compared to E− plants, *E*.* pampeana* provided more benefits to the biomass production of the host plants compared to *E*.* tembladerae*. Hamilton et al. (2010) also found that *Festuca arizonica* plants, harboring *E*.* tembladerae* or *E*.* huerfana*, showed different biomass production under all treatments. However, studies of *H*.* europaeus* indicated that *E*.* hordelymi*,* E*.* sylvatica* ssp. *Pollinensis,* and *E*.* bromicola* did not show different effects on the vegetative growth of host plants (Oberhofer & Leuchtmann, 2014).

In this study, plants harboring *Eg* showed significantly higher aboveground biomass than *Es*‐infected plants under HN conditions. On the other hand, endophyte species did not show any influence on *P*_max_, PNUE, LMA, or the total phenolic concentrations of *A*.* sibiricum* plants, which is related to plant defense. It has been reported that plants infected with *E*.* pampeana* showed a significantly higher tiller number than plants infected with *E*.* tembladerae* (Iannone et al., 2012). Furthermore, loline alkaloids were only detected in host plants harboring *E*.* pampeana* (Iannone et al., 2012). The lack of any significant concentrations of the endophyte alkaloids ergovaline and peramine suggests that these endophyte alkaloids do not play a role in the benefits of endophyte infection on the *A*.* sibiricum* host at the present stage. Considering that the difference only occurred in the HN treatment, we suspect that the growth characteristics of host plants harboring different endophytes may vary due to different levels of nitrogenous compounds (e.g., total soluble protein and total amino acids; Ryan, Rasmussen, Xue, Parsons, & Newman, 2014) or phytohormones (e.g., indole‐3‐acetic acid (IAA) and gibberellin (GA); De Battista, Bacon, Severson, Plattner, & Bouton, 1990; Latch, Hunt, & Musgrave, 1985) produced or induced by the endophytes.

The symbiotic relationships between plants and fungi have a long evolutionary history. Illuminating what determines the diversity and structure of natural communities has long been a research hot spot (Jani et al., 2010). In the present study, plants harboring *Eg* showed significantly higher aboveground biomass than *Es*‐infected plants under HN conditions, but not under LN conditions. Phoenix et al. (2012) proposed that nitrophilous species may be more competitive with increased nitrogen deposition and are then able to exclude other plant species in the habitat. As with most coevolutionary interactions, *A. sibiricum* that harbored *Eg* and *Es* may show pronounced geographic variation in natural habitats in the future and may change the diversity of natural communities (Cheplick & Faeth 2009).

### 4.2 Does the host maternal genotype affect the symbiosis of endophytes and native grass?

The genetic variation of the host plant, even in cultivated grasses, where host genetic variation is reduced relative to wild grasses because of cultivation and selective breeding, can also result in different growth performances (Cheplick & Cho, 2003). Studies using *L*.* perenne* suggested that the plant genotype and its interaction with endophyte infection influences many traits of host plants, such as vegetative growth (Cheplick, 2004; Cheplick & Cho, 2003), physiological characteristics of photosynthesis (Spiering, Greer, & Schmid, 2006), biomass production (Cheplick, 2004, 2007; Hesse et al., 2004), and reproductive growth (Hesse et al., 2004). Research on native grass–endophyte symbiosis also supported this view. Faeth and Fagan (2002) conducted a field experiment using *Festuca arizonica* and suggested that the plant maternal genotype interacted with endophyte infection to affect the growth rate of host plants. After a growing season, two genotypes of E+ plants showed a greater growth rate than that of E− plants, while the others were not affected by endophyte infection. In another study of the same grass species (the same four genotypes) lasting 2 years in the field, the genotype × endophyte interaction also had significant effects on the growth of the host plants. However, in this study, endophyte infection showed negative effects in three genotype hosts (Faeth et al., 2002). Similarly, in the present study, the plant maternal genotype significantly influenced the *P*_max_ and shoot biomass of *A*.* sibiricum*. In addition, endophyte infection appears to override the host genotype, at least in determining the total phenolic concentration, LMA, and several photosynthetic parameters of *A*.* sibiricum* plants.

### 4.3 Does the influence of endophyte species depend on nitrogen availability?

Recent studies showed that the potential effect of endophyte infection on host performance can also be influenced by the nitrogen conditions in the environment (Ahlholm, Helander, Lehtimäki, Wäli, & Saikkonen, 2002; Cheplick, 1998; Saikkonen et al., 2006). Saikkonen et al. (2006) conducted a meta‐analysis of the primary literature and demonstrated that the positive effects of endophytes appear to be dependent on high nutrient availability in soils. In the present study, we found that the effect of endophyte infection on host plants varied between HN and LN groups. Under HN conditions, E+ plants performed better than E− plants in terms of aboveground biomass, which is consistent with the results obtained in tall fescue and *L*.* perenne* (Ren et al., 2009; Saikkonen et al., 2006). Under LN conditions, this positive effect was weaker, while E+ plants did show a higher aboveground biomass than E− plants. This was similar to a previous study using *A. sibiricum* (Li Ren, Han, Yin, Wei, & Gao 2012). Under nitrogen deficiency, the N concentration was lower for E+ compared to E− plants; however, E+ plants had an elevated photosynthetic capacity. *Achnatherum sibiricum* plants with a higher PNUE could be the result of investing relatively more of their N in photosynthetic machinery (Li et al. 2012). Previous reports have also indicated that organisms with a greater growth advantage in nutrient‐poor conditions are those that are able to modify their body nutrient content and increase the efficiency of nutrient use without major decreases in their growth rates (Elser et al., 2003; Mulder & Bowden, 2007). Therefore, E+ plants grew better than E− plants by lowering their N concentrations while increasing their PNUE and *P*_max_ under LN conditions. Certainly, the medium in which *A*.* sibiricum* grew in the LN treatment was consistent with the nitrogen content of natural habitats and not extremely nitrogen poor. If the nitrogen supply is extremely poor, the beneficial effect of endophyte infection might not occur (Cheplick, Clay, & Marks, 1989; Newman et al., 2003). Wan (2006) established a field experiment to examine the ecosystem response to N enrichment in Inner Mongolia grassland. The biomass and dominance of *A. sibiricum* increased significantly with higher rates of nitrogen addition. The frequency of *Epichloë* endophyte infections in natural grass populations has often been used to infer relative fitness advantages of harboring the endophytes (Cheplick, 1998; Clay, 1998). Higher frequencies of endophyte infection were thought to be reflective of greater fitness advantages over E− plants. In the present study, the higher beneficial effect of endophyte infection on host plants under HN conditions may indicate the higher endophyte infection frequency of *A*.* sibiricum* in natural habitats with increased nitrogen deposition.

### 4.4 Is the effect of stroma‐bearing endophytes on host plants is negative?

It has been reported that endophytes that reproduce solely by vertical transmission should have reduced virulence to maintain the productivity of the host plants (Ewald, 1987, 1993). Therefore, symbioses of grasses with vertically transmitted endophytes may be highly mutualistic because both host and endophyte contribute to the persistence of the association (Ewald, 1987, 1993). However, endophyte species with a sexual cycle can produce stromata on flowering tillers, which prevents the floral development and seed set of the plant. These endophytes develop disease symptoms during their sexual cycles, and negative effects on host plants may predominate (Clay & Schardl, 2002; Ewald, 1987, 1993; Herre, 1993; Toft, Aeschlimann, & Bolis, 1991). A 3‐year field experiment conducted by Zabalgogeazcoa, Ciudad, Leuchtmann, Vázquez de Aldana, and Criado (2008), however, showed that endophytes causing choke disease may not have a negative effect during their vegetative growth stage. The results showed that E+ *Brachypodium phoenicoides* plants showed no significant differences in biomass production compared to E− plants during their vegetative growth stage. In addition, Groppe et al. (1999) found that *E*.* bromicola*, which causes choke disease in *B*.* erectus*, even increased the vegetative tillering and aboveground biomass of host plants. In the present study, *A*.* sibiricum* plants harbored one of two different types of endophyte (*Es* and *Eg*). Although *Eg* and *Es* are vertically transmitted, plants harboring *Eg* can occasionally produce conidia stromata and may be horizontally transmitted (Li et al., 2015). The results in our study showed that *Eg‐*infected plants had significantly higher biomass production than *Es‐*infected hosts. Using *A*.* sibiricum* plants harboring either a vertically transmitted or vertically and horizontally transmitted endophyte, the results in our study confirmed the positive effects of a stroma‐bearing endophyte on host plants during their vegetative growth stage.

### 4.5 Possible evolutionary development of *E. gansuensis* and *E. sibirica*

In natural grass populations, the presence of *E. gansuensis* in both *A. sibiricum* and *Achnatherum inebrians* is common (Li et al., 2004; Zhang et al., 2009). *Achnatherum sibiricum* and *A. inebrians* belong to the same genus, are distributed in adjacent regions, and even share certain areas (Ma, 1985; Shi, 1997). However, *E. gansuensis* exhibits different abilities for alkaloid production in these two hosts. *Achnatherum inebrians* has been reported to possess ergonovine and ergine, which can cause toxicity in livestock (Li et al., 2004). In contrast, *A. sibiricum* has no obvious herbivore deterrence (Jin & Han, 2010). In the present study, the lack of any significant concentrations of ergovaline and peramine was observed in *A. sibiricum* that harbored *Eg*. For that matter, *Eg* exhibits greater beneficial effects in *A. inebrians* than in *A. sibiricum*. In addition, *Eg* inhibits a different transmission mode in these two hosts. *Achnatherum sibiricum‐* and *A. inebrians‐*harboring *Eg* can be vertically transmitted, while *A. sibiricum* plants harboring *Eg* can occasionally produce conidia stromata and may be horizontally transmitted (Li et al., 2015). Many mutualistic interactions are thought to undergo the evolutionary pathway from pathogenic toward mutualistic symbioses. In this regard, the coevolution of endophytes with host plants may include the complete loss of horizontal transmission and sexual reproduction as well as the complete dependency of the host (Gundel, Omacini, Sadras, & Ghersa, 2010). It is possible that *A. sibiricum* may have been infected by *Eg* earlier than *Es*, and the beneficial effect of *Eg* to *A. sibiricum* was mainly in growth enhancement. When the host grass was exposed to a stressful environment such as low precipitation, infection with *Es* might be improved (Zhang et al., 2009). Thus, we speculated that changes in the contribution of *Eg* and *Es* to the host might occur under different environmental conditions.

Endophytic fungi are important but relatively unstudied microbial plant symbionts. It has been reported that plant survival and distribution can be influenced by endophytic communities (Qadri, Rajput, Abdin, Vishwakarma, & Riyaz‐Ul‐Hassan, 2014). However, results from studies using agronomic grass relative to natural populations and communities may be too simplistic (Saikkonen et al., 2006). With the greater genetic diversity in both the endophyte (Faeth & Sullivan, 2003) and its grass host (Saikkonen et al., 2006), natural populations should exhibit much greater variation. In the present study, we found the outcomes of endophyte *A. sibiricum* symbiosis in terms of host biomass and photosynthesis rates greatly depend on both environmental conditions and host endophyte genotypic combinations.

## Conflict of interest

The authors declare that they have no conflict of interest.

## Ethical approval

This article does not contain any studies with human participants or animals performed by any of the authors.

## References

[ece32566-bib-0001] Ahlholm, J. U. , Helander, M. , Lehtimäki, S. , Wäli, P. , & Saikkonen, K. (2002). Vertically transmitted fungal endophytes: Different responses of host‐parasite systems to environmental conditions. Oikos, 99, 173–183.

[ece32566-bib-0111] Amalric, C. , Sallanon, H. , Monnet, F. , Hitmi, A. , Coudret, A. , & Amalric, C. (1999). Gas exchanges and fluorescence parameters of symbiotic and non symbiotic rye‐grass under water stress. Photosynthetica, 37, 107–112.

[ece32566-bib-0002] Arachevaleta, M. , Bacon, C. W. , Hoveland, C. S. , & Radcliffe, D. E. (1989). Effect of the tall fescue endophyte on plant response to environmental stress. Agronomy Journal, 81, 83–90.

[ece32566-bib-0003] Assuero, S. G. , Matthew, C. , Kemp, P. D. , Latch, G. C. M. , Barker, D. J. , & Haslett, S. J. (2000). Morphological and physiological effects of water deficit and endophyte infection on contrasting tall fescue cultivars. New Zealand Journal of Agricultural Research, 43, 49–61.

[ece32566-bib-0004] Augé, R. M. , Kubikova, E. , & Moore, J. L. (2001). Foliar dehydration tolerance of mycorrhizal cowpea, soybean and bush bean. New Phytologist, 151, 535–541.

[ece32566-bib-0112] Belesky, D. P. , Devine, O. J. , Pallas, J. E. . Jr , & Stringer, W.C. (1987). Photosynthetic activity of tall fescue as influenced by a fungal endophyte. Photosynthetica, 21, 82–87.

[ece32566-bib-0005] Belesky, D. P. , & Fedders, J. M. (1996). Does endophyte influence regrowth of tall fescue? Annals of Botany (London), 78, 499–505.

[ece32566-bib-0006] Blankenship, J. D. , Spiering, M. J. , Wilkinson, H. H. , Fannin, F. F. , Bush, L. P. , & Schardl, C. L. (2001). Production of loline alkaloids by the grass endophyte, *Neotyphodium uncinatum*, in defined media. Phytochemistry, 58, 395–401. 11557071 10.1016/s0031-9422(01)00272-2

[ece32566-bib-0007] Brem, D. , & Leuchtmann, A. (2001). *Epichloë* grass endophytes increase herbivore resistance in the woodland grass *Brachypodium sylvaticum* . Oecologia, 126, 522–530. 10.1007/s004420000551 28547237

[ece32566-bib-0008] Chen, G. Y. , Yu, G. L. , Chen, Y. , & Xu, D. Q. (2006). Exploring the observation methods of photosynthetic responses to light and carbon dioxide. Journal of Plant Physiology and Molecular Biology, 32, 691–696. 17167207

[ece32566-bib-0009] Cheplick, G. P. (1997). Effects of endophytic fungi on the phenotypic plasticity of *Lolium perenne* (Poaceae). American Journal of Botany, 84, 34–40.

[ece32566-bib-0010] Cheplick, G. P. (1998). Genotypic variation in the regrowth of *Lolium perenne* following clipping: Effects of nutrients and endophytic fungi. Functional Ecology, 12, 176–184.

[ece32566-bib-0011] Cheplick, G. P. (2004). Recovery from drought stress in *Lolium perenne* (Poaceae): Are fungal endophytes detrimental. American Journal of Botany, 91, 1960–1968. 21652344 10.3732/ajb.91.12.1960

[ece32566-bib-0012] Cheplick, G. P. (2007). Costs of fungal endophyte infection in *Lolium perenne* genotypes from Eurasia and North Africa under extreme resource limitation. Environmental and Experimental Botany, 60, 202–210.

[ece32566-bib-0013] Cheplick, G. P. (2008). Host genotype overrides fungal endophyte infection in influencing tiller and spike production of *Lolium perenne* (Poaceae) in a common garden experiment. American Journal of Botany, 95, 1063–1071. 21632426 10.3732/ajb.0800042

[ece32566-bib-0014] Cheplick, G. P. , & Cho, R. (2003). Interactive effects of fungal endophyte infection and host genotype on growth and storage in *Lolium perenne* . New Phytologist, 158, 183–191.

[ece32566-bib-0015] Cheplick, G. P. , Clay, K. , & Marks, S. (1989). Interactions between infection by endophytic fungi and nutrient limitation in the grasses *Lolium perenne* and *Festuca arundinacea* . New Phytologist, 111, 89–97.

[ece32566-bib-0113] Cheplick, G. P. , & Faeth, S. H. (2009). Ecology and evolution of the grass‐endophyte symbiosis. Oxford, New York: Oxford University Press.

[ece32566-bib-0016] Christensen, M. J. , & Bennett, R. J. (2002). Growth of *Epichloë*/*Neotyphodium* and p‐endophytes in leaves of *Lolium* and *Festuca* grasses. Mycological Research, 106(1), 93–106.

[ece32566-bib-0017] Clay, K. (1990). Fungal endophytes of grasses. Annual Review of Ecology and Systematics, 21, 275–297.

[ece32566-bib-0018] Clay, K. (1998). Fungal endophyte infection and the population dynamics of grasses In Cheplick G. P. (Ed.), The population biology of grasses (pp. 255–285). Cambridge: Cambridge University Press.

[ece32566-bib-0019] Clay, K. , & Schardl, C. L. (2002). Evolutionary origins and ecological consequences of endophyte symbiosis with grasses. American Naturalist, 160, S99–S127. 10.1086/342161 18707456

[ece32566-bib-0020] Crawford, N. M. (1995). Nitrate: Nutrient and signal for plant growth. The Plant Cell, 7, 859–868. 7640524 10.1105/tpc.7.7.859 PMC160877

[ece32566-bib-0021] Crawford, K. M. , Land, J. M. , & Rudgers, J. A. (2010). Fungal endophytes of native grasses decrease insect herbivore preference and performance. Oecologia, 164, 431–444. 20585809 10.1007/s00442-010-1685-2

[ece32566-bib-0022] De Battista, J. P. , Bacon, C. W. , Severson, R. , Plattner, R. D. , & Bouton, J. H. (1990). Indole acetic acid production by the fungal endophyte of tall fescue. Agronomy Journal, 82, 878–880.

[ece32566-bib-0023] De‐wen, T. , Wang, J. , Patchett, B. , & Gooneratne, R. (2006). Seasonal change of loline alkaloids in endophyte infected meadow fescue. Agricultural Sciences in China, 5, 793–797.

[ece32566-bib-0024] Dighton, J. , White, J. F. Jr , & Oudemans, P. (2005). The fungal community: Its organization and role in the ecosystem. Boca Raton: CRC Press.

[ece32566-bib-0025] Dreyfuss, M. M. , & Chapela, I. H. (1994). Potential of fungi in discovery of novel low molecular weight pharmaceuticals In Gullo V. P. (Ed.), The discovery of natural products with therapeutic potential (pp. 49–80). London, UK: Butterworth‐Heinemann. 10.1016/b978-0-7506-9003-4.50009-5 7749314

[ece32566-bib-0026] Elser, J. J. , Acharya, K. , Kyle, M. , Cotner, J. , Makino, W. , Markow, T. , … Sterner, R. W. (2003). Growth rate‐stoichiometry couplings in diverse biota. Ecology Letters, 6, 936–943.

[ece32566-bib-0027] Ewald, P. W. (1987). Transmission modes and evolution of the parasitism‐mutualism continuuma. Annals of the New York Academy of Sciences, 503, 295–306. 3304078 10.1111/j.1749-6632.1987.tb40616.x

[ece32566-bib-0028] Ewald, P. W. (1993). Evolution of infectious disease. Oxford: Oxford University Press.

[ece32566-bib-0029] Faeth, S. H. , Bush, L. P. , & Sullivan, T. J. (2002). Peramine alkaloid variation in *Neotyphodium*‐infected Arizona fescue: Effects of endophyte and host genotype and environment. Journal of Chemical Ecology, 28, 1511–1526. 12371807 10.1023/a:1019916227153

[ece32566-bib-0030] Faeth, S. H. , & Fagan, W. F. (2002). Fungal endophytes: Common host plant symbionts but uncommon mutualists. Integrative and Comparative Biology, 42, 360–368. 21708729 10.1093/icb/42.2.360

[ece32566-bib-0031] Faeth, S. H. , Helander, M. L. , & Saikkonen, K. T. (2004). Asexual *Neotyphodium* endophytes in a native grass reduce competitive abilities. Ecology Letters, 7, 304–313.

[ece32566-bib-0032] Faeth, S. H. , & Sullivan, T. J. (2003). Mutualistic asexual endophytes in a native grass are usually parasitic. The American Naturalist, 161, 310–325. 10.1086/345937 12675375

[ece32566-bib-0033] Franche, C. , Lindström, K. , & Elmerich, C. (2009). Nitrogen‐fixing bacteria associated with leguminous and non‐leguminous plants. Plant and Soil, 321, 35–39.

[ece32566-bib-0034] Gibert, A. , & Hazard, L. (2013). Genetically based vertical transmission drives the frequency of the symbiosis between grasses and systemic fungal endophytes. Journal of Ecology, 101, 743–752.

[ece32566-bib-0035] Glenn, A. E. , Bacon, C. W. , Price, R. , & Hanlin, R. T. (1996). Molecular phylogeny of *Acremonium* and its taxonomic implications. Mycologia, 88, 369–383.

[ece32566-bib-0036] Groppe, K. , Steinger, T. , Sanders, I. , Schmid, B. , Wiemken, A. , & Boller, T. (1999). Interaction between the endophytic fungus *Epichloë bromicola* and the grass *Bromus erectus*: Effects of endophyte infection, fungal concentration and environment on grass growth and flowering. Molecular Ecology, 8, 1827–1835. 10620227 10.1046/j.1365-294x.1999.00772.x

[ece32566-bib-0037] Gundel, P. E. , Helander, M. , Casas, C. , Hamilton, C. E. , Faeth, S. H. , & Saikkonen, K. (2013). *Neotyphodium* fungal endophyte in tall fescue (*Schedonorus phoenix*): A comparison of three Northern European wild populations and the cultivar kentucky‐31. Fungal Diversity, 60, 15–24.

[ece32566-bib-0038] Gundel, P. E. , Omacini, M. , Sadras, V. O. , & Ghersa, C. M. (2010). The interplay between the effectiveness of the grass‐endophyte mutualism and the genetic variability of the host plant. Evolutionary Applications, 3, 538–546. 25567945 10.1111/j.1752-4571.2010.00152.x PMC3352510

[ece32566-bib-0039] Hamilton, C. E. , Dowling, T. E. , & Faeth, S. H. (2010). Hybridization in endophyte symbionts alters host response to moisture and nutrient treatments. Microbial Ecology, 59, 768–775. 19921327 10.1007/s00248-009-9606-9

[ece32566-bib-0040] Hamilton, C. E. , & Faeth, S. H. (2005). Asexual *Neotyphodium* endophytes in Arizona fescue: A test of the seed germination and pathogen resistance hypothesis. Symbiosis, 38, 69–85.

[ece32566-bib-0041] Hardoim, P. R. , van Overbeek, L. S. , & van Elsas, J. D. (2008). Properties of bacterial endophytes and their proposed role in plant growth. Trends in Microbiology, 16, 463–471. 18789693 10.1016/j.tim.2008.07.008

[ece32566-bib-0042] Herre, E. A. (1993). Population structure and the evolution of virulence in nematode parasites of fig wasps. Science, 259, 1442–1445. 17801279 10.1126/science.259.5100.1442

[ece32566-bib-0043] Hesse, U. , Hahn, H. , Andreeva, K. , Förster, K. , Warnstorff, K. , Schöberlein, W. , & Diepenbrock, W. (2004). Investigations on the influence of *Neotyphodium* endophytes on plant growth and seed yield of *Lolium perenne* genotypes. Crop Science, 44, 1689–1695.

[ece32566-bib-0044] Hesse, U. , Schöberlein, W. , Wittenmayer, L. , Förster, K. , Warnstorff, K. , Diepenbrock, W. , & Merbach, W. (2003). Effects of *Neotyphodium* endophytes on growth, reproduction and drought‐stress tolerance of three *Lolium perenne* L. genotypes. Grass and Forage Science, 58, 407–415.

[ece32566-bib-0045] Hikosaka, K. (2004). Interspecific difference in the photosynthesis–nitrogen relationship: Patterns, physiological causes, and ecological importance. Journal of Plant Research, 117, 481–494. 15583974 10.1007/s10265-004-0174-2

[ece32566-bib-0046] Höft, M. , Verpoorte, R. , & Beck, E. (1996). Growth and alkaloid contents in leaves of *Tabernaemontana pachysiphon* Stapf (Apocynaceae) as influenced by light intensity, water and nutrient supply. Oecologia, 107, 160–169. 10.1007/BF00327899 28307301

[ece32566-bib-0047] Iannone, L. J. , Pinget, A. D. , Nagabhyru, P. , Schardl, C. L. , & De Battista, J. P. (2012). Beneficial effects of *Neotyphodium tembladerae* and *Neotyphodium pampeanum* on a wild forage grass. Grass and Forage Science, 67, 382–390.

[ece32566-bib-0048] Jani, A. J. , Faeth, S. H. , & Gardner, D. (2010). Asexual endophytes and associated alkaloids alter arthropod community structure and increase herbivore abundances on a native grass. Ecology Letters, 13, 106–117. 19912292 10.1111/j.1461-0248.2009.01401.x

[ece32566-bib-0049] Jin, X. M. , & Han, G. D. (2010). Effects of grazing intensity on species diversity and structure of meadow steppe community. Pratacultural Science (in Chinese), 27, 7–10.

[ece32566-bib-0050] Latch, G. C. M. , Christensen, M. J. , & Samuels, G. J. (1984). Five endophytes of Lolium and Festuca in New Zealand [fungi, description, new taxa]. Mycotaxon.

[ece32566-bib-0051] Latch, G. C. M. , Hunt, W. F. , & Musgrave, D. R. (1985). Endophytic fungi affect growth of perennial ryegrass. New Zealand Journal of Agricultural Research, 28, 165–168.

[ece32566-bib-0052] Leuchtmann, A. , & Oberhofer, M. (2013). The *Epichloë* endophytes associated with the woodland grass *Hordelymus europaeus* including four new taxa. Mycologia, 105, 1315–1324. 23921239 10.3852/12-400

[ece32566-bib-0053] Lewis, G. C. (2004). Effects of biotic and abiotic stress on the growth of three genotypes of *Lolium perenne* with and without infection by the fungal endophyte *Neotyphodium lolii* . Annals of Applied Biology, 144, 53–63.

[ece32566-bib-0054] Li, X. , Han, R. , Ren, A. Z. , & Gao, Y. B. (2010). Using high‐temperature treatment to construct endophyte‐free *Achnatherum sibiricum* . Microbiology China, 37, 1395–1400.

[ece32566-bib-0055] Li, C. J. , Nan, Z. B. , Paul, V. H. , Dapprich, P. D. , & Liu, Y. (2004). A new *Neotyphodium* species symbiotic with drunken horse grass (*Achnatherum inebrians*) in China. Mycotaxon, 90, 141–147.

[ece32566-bib-0114] Li, X. , Ren, A. Z. , Han, R. , Yin, L. J. , Wei, M. Y. , & Gao, Y. B. (2012). Endophyte‐mediated effects on the growth and physiology of Achnatherum sibiricum are conditional on both N and P availability. Plos One, 7(11), e48010. 23185245 10.1371/journal.pone.0048010 PMC3502411

[ece32566-bib-0056] Li, X. , Zhou, Y. , Zhu, M. J. , Qin, J. H. , Ren, A. Z. , & Gao, Y. B. (2015). Stroma‐bearing endophyte and its potential horizontal transmission ability in *Achnatherum sibiricum* . Mycologia, 107, 21–31. 25344262 10.3852/13-355

[ece32566-bib-0057] Liu, H. F. (2004). Eco‐genetic analysis on the variation and differentiation of Leymus chinensis populations in mid‐eastern Inner Mongolia Steppe. Tianjin, China: Nankai University Press.

[ece32566-bib-0058] Ma, Y. Q. (1985). Flora Inner Mongolia. Hohhot: Inner Mongolia People's Press.

[ece32566-bib-0059] Malinowski, D. P. , Belesky, D. P. , Hill, N. S. , Baligar, V. C. , & Fedders, J. M. (1998). Influence of phosphorus on the growth and ergot alkaloid content of *Neotyphodium coenophialum*‐infected tall fescue (*Festuca arundinacea* Schreb.). Plant and Soil, 198, 53–61.

[ece32566-bib-0060] Marks, S. , & Clay, K. (1996). Physiological responses of *Festuca arundinacea* to fungal endophyte infection. New Phytologist, 133, 727–733.

[ece32566-bib-0061] Marschner, H. (1995). Mineral nutrition of higher plants. London: Academic Press.

[ece32566-bib-0062] Moon, C. D. , Guillaumin, J. J. , Ravel, C. , Li, C. J. , Craven, K. D. , & Schardl, C. L. (2007). New *Neotyphodium* endophyte species from the grass tribes Stipeae and Meliceae. Mycologia, 99, 895–905. 18333513 10.3852/mycologia.99.6.895

[ece32566-bib-0063] Moore, J. R. , Pratley, J. E. , Mace, W. J. , & Weston, L. A. (2015). Variation in alkaloid production from genetically diverse *Lolium* accessions infected with *Epichloë* species. Journal of Agricultural and Food Chemistry, 63, 10355–10365. 26550846 10.1021/acs.jafc.5b03089

[ece32566-bib-0064] Morse, L. J. , Day, T. A. , & Faeth, S. H. (2002). Effect of *Neotyphodium* endophyte infection on growth and leaf gas exchange of Arizona fescue under contrasting water availability regimes. Environmental and Experimental Botany, 48, 257–268.

[ece32566-bib-0065] Morse, L. J. , Faeth, S. H. , & Day, T. A. (2007). *Neotyphodium* interactions with a wild grass are driven mainly by endophyte haplotype. Functional Ecology, 21, 813–822.

[ece32566-bib-0066] Mulder, K. , & Bowden, W. B (2007). Organismal stoichiometry and the adaptive advantage of variable nutrient use and production efficiency in *Daphnia* . Ecological Modelling, 202, 427–440.

[ece32566-bib-0067] Nan, Z. B. (1996). Effects of *Acremonium* endophyte on the growth of *Hordeum bodganii* . Pratacultural Science, 13, 16–18.

[ece32566-bib-0068] Newman, J. A. , Abner, M. L. , Dado, R. G. , Gibson, D. J. , Brookings, A. , & Parsons, A. J. (2003). Effects of elevated CO_2_: Nitrogen and fungal endophyte‐infection on tall fescue: Growth, photosynthesis, chemical composition and digestibility. Global Change Biology, 9, 425–437.

[ece32566-bib-0069] Oberhofer, M. , & Leuchtmann, A. (2014). Horizontal transmission, persistence and competition capabilities of *Epichloë* endophytes in *Hordelymus europaeus* grass hosts using dual endophyte inocula. Fungal Ecology, 11, 37–49.

[ece32566-bib-0070] Phoenix, G. K. , Emmett, B. A. , Britton, A. J. , Caporn, S. J. M. , Dise, N. B. , Helliwell, R. , … Power, S. A. (2012). Impacts of atmospheric nitrogen deposition: Responses of multiple plant and soil parameters across contrasting ecosystems in long‐term field experiments. Global Change Biology, 18, 1197–1215.

[ece32566-bib-0071] Qadri, M. , Rajput, R. , Abdin, M. Z. , Vishwakarma, R. A. , & Riyaz‐Ul‐Hassan, S. (2014). Diversity, molecular phylogeny, and bioactive potential of fungal endophytes associated with the Himalayan blue pine (*Pinus wallichiana*). Microbial Ecology, 67, 877–887. 24563192 10.1007/s00248-014-0379-4

[ece32566-bib-0072] Rahman, M. H. , & Saiga, S. (2005). Endophytic fungi (*Neotyphodium coenophialum*) affect the growth and mineral uptake, transport and efficiency ratios in tall fescue (*Festuca arundinacea*). Plant and Soil, 272, 163–171.

[ece32566-bib-0073] Ravel, C. , Courty, C. , Coudret, A. , & Charmet, G. (1997). Beneficial effects of *Neotyphodium lolii* on the growth and the water status in perennial ryegrass cultivated under nitrogen deficiency or drought stress. Agronomie, 17, 173–181.

[ece32566-bib-0074] Ren, A. Z. , Gao, Y. B. , Wang, W. , Wang, J. L. , & Zhao, N. X. (2009). Influence of nitrogen fertilizer and endophyte infection on ecophysiological parameters and mineral element content of perennial ryegrass. Journal of integrative plant biology, 51, 75–83. 19166497 10.1111/j.1744-7909.2008.00721.x

[ece32566-bib-0075] Ren, A. Z. , Li, X. , Han, R. , Yin, L. J. , Wei, M. Y. , & Gao, Y. B. (2011). Benefits of a symbiotic association with endophytic fungi are subject to water and nutrient availability in *Achnatherum sibiricum* . Plant and Soil, 346, 363–373.

[ece32566-bib-0076] Rodriguez, R. J. , White, J. F. Jr. , Arnold, A. E. , & Redman, R. S. (2009). Fungal endophytes: Diversity and functional roles. New Phytologist, 182, 314–330. 19236579 10.1111/j.1469-8137.2009.02773.x

[ece32566-bib-0077] Rostás, M. , Cripps, M. G. , & Silcock, P. (2015). Aboveground endophyte affects root volatile emission and host plant selection of a belowground insect. Oecologia, 177, 487–497. 25284612 10.1007/s00442-014-3104-6

[ece32566-bib-0078] Rúa, M. A. , McCulley, R. L. , & Mitchell, C. E. (2013). Fungal endophyte infection and host genetic background jointly modulate host response to an aphid‐transmitted viral pathogen. Journal of Ecology, 101, 1007–1018.

[ece32566-bib-0079] Rudgers, J. A. , Koslow, J. M. , & Clay, K. (2004). Endophytic fungi alter relationships between diversity and ecosystem properties. Ecology Letters, 7, 42–51.

[ece32566-bib-0080] Rudgers, J. A. , & Swafford, A. L. (2009). Benefits of a fungal endophyte in *Elymus virginicus* decline under drought stress. Basic and Applied Ecology, 10, 43–51.

[ece32566-bib-0081] Ryan, G. D. , Rasmussen, S. , Xue, H. , Parsons, A. J. , & Newman, J. A. (2014). Metabolite analysis of the effects of elevated CO_2_ and nitrogen fertilization on the association between tall fescue (*Schedonorus arundinaceus*) and its fungal symbiont *Neotyphodium coenophialum* . Plant, Cell and Environment, 37, 204–212. 10.1111/pce.12146 23742115

[ece32566-bib-0082] Saari, S. , Helander, M. , Faeth, S. H. , & Saikkonen, K. (2010). The effects of endophytes on seed production and seed predation of tall fescue and meadow fescue. Microbial Ecology, 60, 928–934. 20871988 10.1007/s00248-010-9749-8

[ece32566-bib-0083] Saikkonen, K. , Helander, M. , Faeth, S. H. , Schulthess, F. , & Wilson, D. (1999). Endophyte‐grass‐herbivore interactions: The case of *Neotyphodium* endophytes in Arizona fescue populations. Oecologia, 121, 411–420. 10.1007/s004420050946 28308331

[ece32566-bib-0084] Saikkonen, K. , Lehtonen, P. , Helander, M. , Koricheva, J. , & Faeth, S. H. (2006). Model systems in ecology: Dissecting the endophyte‐grass literature. Trends in Plant Science, 11, 428–433. 16890473 10.1016/j.tplants.2006.07.001

[ece32566-bib-0086] Schardl, C. L. , Leuchtmann, A. , & Spiering, M. J. (2004). Symbioses of grasses with seedborne fungal endophytes. Annual Review of Plant Biology, 55, 315–340. 10.1146/annurev.arplant.55.031903.141735 15377223

[ece32566-bib-0087] Shi, Z. C. (1997). Important poisonous plants of China grassland. Beijing: Agriculture Press.

[ece32566-bib-0088] Shukla, K. , Hager, H. A. , Yurkonis, K. A. , & Newman, J. A. (2015). Effects of the *Epichloë* fungal endophyte symbiosis with *Schedonorus pratensis* on host grass invasiveness. Ecology and Evolution, 5, 2596–2607. 26257873 10.1002/ece3.1536 PMC4523356

[ece32566-bib-0089] Shymanovich, T. , Saari, S. , Lovin, M. E. , Jarmusch, A. K. , Jarmusch, S. A. , Musso, A. M. , … Faeth, S. H. (2015). Alkaloid variation among Epichloid endophytes of sleepygrass (*Achnatherum robustum*) and consequences for resistance to insect herbivores. Journal of Chemical Ecology, 41, 93–104. 25501262 10.1007/s10886-014-0534-x

[ece32566-bib-0090] Smith, S. E. , & Read, D. J. (2008). Mycorrhizal symbiosis, 3rd ed. New York: Academic Press.

[ece32566-bib-0091] Spiering, M. J. , Greer, D. H. , & Schmid, J. (2006). Effects of the fungal endophyte, *Neotyphodium lolii*, on net photosynthesis and growth rates of perennial ryegrass (*Lolium perenne*) are independent of in planta endophyte concentration. Annals of Botany, 98, 379–387. 16735403 10.1093/aob/mcl108 PMC2803460

[ece32566-bib-0092] Stitt, M. , & Krapp, A. (1999). The interaction between elevated carbon dioxide and nitrogen nutrition: The physiological and molecular background. Plant, Cell and Environment, 22, 583–621.

[ece32566-bib-0093] Sullivan, T. J. , & Faeth, S. H. (2004). Gene flow in the endophyte *Neotyphodium* and implications for coevolution with *Festuca arizonica* . Molecular Ecology, 13, 649–656. 14871368 10.1046/j.1365-294x.2004.02091.x

[ece32566-bib-0094] Toft, C. A. , Aeschlimann, A. , & Bolis, L. (1991). Parasite–host associations continued. Oxford: Oxford University Press.

[ece32566-bib-0095] Wan, H. W. (2006). Responses of plant traits and soil microbial biomass C, N, P to nitrogen addition in mature and degraded Leymus chinensis steppe ecosystems in Inner Mongolia plateau. Beijing: The Chinese Academy of Sciences press.

[ece32566-bib-0096] Wei, Y. K. , Gao, Y. B. , Li, C. , Xu, H. , & Ren, A. Z. (2006). Genetic diversity of *Neotyphodium* endophytes isolated from *Achnatherum sibiricum* populations in mid‐ and eastern Inner Mongolia steppe, China. Zhiwu Shengtai Xuebao, 30, 640–649.

[ece32566-bib-0097] West, C. P. , Popay, A. J. , & Thom, E. R. (2007). Plant influences on endophyte expression. Proceedings of the proceedings of the 6th international symposium on fungal endophytes of grasses, pp. 117–121.

[ece32566-bib-0098] Zabalgogeazcoa, I. , Ciudad, A. G. , Leuchtmann, A. , Vázquez de Aldana, B. R. , & Criado, B. G. (2008). Effects of choke disease in the grass *Brachypodium phoenicoides* . Plant Pathology, 57, 467–472.

[ece32566-bib-0099] Zhang, X. X. , Fan, X. M. , Li, C. J. , & Nan, Z. B. (2010). Effects of cadmium stress on seed germination, seedling growth and antioxidative enzymes in *Achnatherum inebrians* plants infected with a *Neotyphodium* endophyte. Plant Growth Regulation, 60, 91–97.

[ece32566-bib-0100] Zhang, X. , Ren, A. Z. , Wei, Y. K. , Lin, F. , Li, C. , Liu, Z. J. , & Gao, Y. B. (2009). Taxonomy, diversity and origins of symbiotic endophytes of *Achnatherum sibiricum* in the Inner Mongolia steppe of China. FEMS Microbiology Letters, 301, 12–20. 19863662 10.1111/j.1574-6968.2009.01789.x

